# Long-term impact of COVID-19 pandemic: Moral tensions, distress, and injuries of healthcare workers

**DOI:** 10.1371/journal.pone.0298615

**Published:** 2024-09-27

**Authors:** Lianne Jeffs, Natalie Heeney, Jennie Johnstone, Jon Hunter, Carla Adrienne Loftus, Leanne Ginty, Rebecca Greenberg, Lesley Wiesenfeld, Robert Maunder

**Affiliations:** 1 Science of Care Institute, Toronto, Ontario, Canada; 2 Sinai Health, Toronto, Ontario, Canada; 3 Department of Psychiatry, University of Toronto, Toronto, Ontario, Canada; 4 Department of Laboratory Medicine and Pathobiology, University of Toronto, Toronto, Ontario, Canada; 5 Department of Psychiatry, Mount Sinai Hospital, Sinai Health, Toronto, Ontario, Canada; 6 Nursing Education and Academic Affairs, Sinai Health, Toronto, Ontario, Canada; 7 Department of Paediatrics, University of Toronto, Toronto, Ontario, Canada; Bangladesh Open University, BANGLADESH

## Abstract

Given the longevity of the COVID-19 pandemic, it is important to address the perceptions and experiences associated with the progression of the pandemic. This narrative can inform future strategies aimed at mitigating moral distress, injury, and chronic stress that restores resilience and well-being of HCWs. In this context, a longitudinal survey design was undertaken to explore how health care workers are experiencing the COVID-19 pandemic over time. A qualitative design was employed to analyze the open ended survey responses using a thematic analysis approach. All physicians and staff at an academic health science centre in Toronto, Ontario, Canada were invited to participate in the survey. The majority of survey respondents were nurses and physicians, followed by researchers/scientists, administrative assistants, laboratory technicians, managers, social workers, occupational therapists, administrators, clerks and medical imaging technologists. The inductive analysis revealed three themes that contributed to moral tensions and injury: 1) experiencing stress and distress with staffing shortages, increased patient care needs, and visitor restrictions; 2) feeling devalued and invisible due to lack of support and inequities; and 3) polarizing anti- and pro-public health measures and incivility. Study findings highlight the spectrum, magnitude, and severity of the emotional, psychological, and physical stress leading to moral injury experienced by the healthcare workforce. Our findings also point to continued, renewed, and new efforts in enhancing both individual and collective moral resilience to mitigate current and prevent future moral tensions and injury.

## Introduction

COVID-19 continues to spread and have global impact, [1–8] having infected over 702 million people and killed over 6.9 million worldwide as of January 30, 2024 [8]. During high incidence peaks, hospitals were overwhelmed with outbreaks and managing severely ill patients [2, 9, 10] amid deepening staffing shortages [10]. The prolonged nature of the pandemic continues to have negative consequences (e.g., death, pain and suffering, burnout) on people’s physical and mental health and well-being [11]. These consequences are intensified for healthcare workers (HCWs) who balanced their need and duty to care [11, 12] within extremely challenging situations and stressful work environments [2, 13–16]. Specifically, HCWs observed and participated in care that was misaligned with what they view to be the best for patients, and witnessed the devastating impact of restricted visitor policies (limiting or no access to loved ones for patient), and missed care [11, 13, 14]. In turn, these tensions in care were experienced as moral dilemmas by HCWs, resulting in increased anxiety, psychological trauma and moral distress [4, 11, 13–15, 17–20]. Further, resolving these moral dilemmas depleted HCWs’ psychological resilience, [21–24] that for some is resulted in moral injury [2, 14, 25–28] and post-traumatic stress [24]. For example, a cross-sectional study reported the overall prevalence of clinically significant moral injury in HCWs of 32.4% [29].

Moral injury occurs when one fails to prevent or commits acts that run contrary to deeply held moral beliefs and expectations, with moral distress being the psychological experience of the inability to execute a morally correct action [14, 27, 30]. Both of these phenomenon were amplified in the context of COVID-19 [25]. For example, one study reported that although moral distress had declined, moral injury remained stable over time in the first year of the pandemic [14]. The majority of evidence regarding the prevalence and impact of moral distress and injury in HCWs during COVID-19 were from cross-sectional, with fewer longitudinal, surveys. Although this provides important data on their incidence and the impact of the pandemic, less is known about the perceptions and experiences over time from a narrative viewpoint. This is an important gap to address, as insights from narrative can provide a richer description of the impact of COVID-19. Specifically, that can inform future strategies aimed at mitigating moral distress, injury, and chronic stress that restores resilience and well-being of HCWs. This paper provides an overview of qualitative findings from open-ended comments provided in a survey administered to healthcare workers in an urban Canadian hospital over seven data points from fall 2020 to spring 2022.

## Methods

### Setting

Sinai Health is an Academic Health Sciences Centre fully affiliated with the University of Toronto located in Toronto, Ontario, that formed in 2015. Sinai Health is comprised of Mount Sinai Hospital, Joseph and Wolf Lebovic Health Complex (442 acute care beds); Hennick Bridgepoint Hospital (464 rehabilitation and complex care beds); Circle of Care (a community based agency); and the Lunenfeld-Tanenbaum Research Institute.

### Study design

As part of a larger survey to study the impact of psychological well-being during COVID-19, two open-ended questions were posed at the end of each survey: “In addition to the specific questions that we ask, what else would you like us to know about you?” and “In addition to the specific questions we ask, what else would you like us to know about working during the COVID-19 pandemic?” A qualitative design was employed to analyze the narrative text provided in response to these two questions. Ethics approval was obtained by the Sinai Health Research Ethics Board (REB approval number: REB# 20-0084-E). Participants provided explicit informed, written, and signed consent to complete the surveys as well as an option to allow comments to be quoted in research reports or not. Surveys were completed online using a PHIPA-compliant software (Alchemer, Louisville, CO).

### Data collection

The survey methods have been described previously with data collected over seven data points [31]. Further details of the different time points is provided in Table 1.

**Table 1:** Survey data collection.

Survey	Approximate Date	Wave or Variant	Further Contextual Factors
*First (T1)*	July 27 –November 15, 2020	Post-Wave 1 of COVID-19 Pandemic	N/A
*Second (T2)*	January 25 –February 15, 2021	Post-Peak of Wave 2 of COVID-19 Pandemic	During provincial Stay at Home order in Toronto. Select HCWs have access to vaccine.
*Third (T3)*	April 25—May 16, 2021	Post-Peak of Wave 3	During provincial Stay at Home order and lockdown across Ontario. Broader vaccine availability.
*Fourth (T4)*	July 26 –August 15, 2021	N/A	Relatively low case numbers
*Fifth (T5)*	October 25 –November 15, 2021	N/A	Relatively low case numbers.
*Sixth (T6)*	January 24 –February 13, 2022	Height of Omicron Wave	N/A
*Seventh (T7)*	April 25 –May 16, 2022	N/A	Provincial Reopening

The first survey was open to all hospital employees, physicians, learners, retail employees, and contractors. It was conducted from July 27-November 15, 2020 (T1)–post-Wave 1 of the COVID-19 pandemic, and during the beginning of Wave 2. The T1 survey was administered in two parts: a pre-survey recruitment phase and a baseline data phase. Participants provided comments during the pre-survey recruitment phase of T1; however, only those who completed the baseline data portion of the T1 survey were used to form the cohort for further follow-up. The second survey was conducted from January 25-February 15, 2021(T2) and took place post peak of Wave 2 of the COVID-19 pandemic, during the provincial Stay at Home order and lockdown in Toronto. The third survey occurred from April 26-May 16, 2021 (T3), following the peak of Wave 3 and again during the provincial Stay at Home order and lockdown across the Ontario. The fourth and fifth surveys were conducted from July 26-August 15, 2021 (T4), and October 25-November 15, 2021 (T5), during periods of relatively low case numbers. The sixth survey occurred from January 24-February 13, 2022 (T6), during the height of the omicron wave, and the final survey was collected from April 25-May 16, 2022 (T7), a period marked by provincial reopening. HCWs had access to the COVID-19 vaccine by T2, with broader availability to any HCW by T3. The qualitative comments utilized in the analysis comes from the same group of 583 participants who received the survey at each time point. Comments for the T1 time point were collected during a pre-survey recruitment phase; however only participants who completed the baseline portion of the T1 survey were used to form the entire survey cohort for further follow-up (n = 583); the entire cohort was sent the survey at each time point.

### Analytical plan

An inductive thematic analysis approach was employed to the narrative datasets (T1-T7) [32, 33]. There were two analytical phases, with the first two narrative datasets (T1, T2) being reviewed independently by four research team members who then convened a series of meetings to review their initial codes. Broader level categories and themes were derived from the initial codes and were populated into a codebook. The second phase involved analyzing the remaining narrative datasets (T3-T7) by a senior researcher. Analysis and refinement of the codebook occurred until there were no new codes or categories emerging from the narrative dataset [34]. To ensure methodological rigor of the final narrative dataset, one of the senior researchers reviewed the codebooks with all the open-ended narrative text from the seven data points.

## Results

### Sample description

The proportion of survey respondents who provided comments and consented to have it shared compared to the number of survey respondents for that time point (out of the total 583 cohort) included: 283/884 (32%) at T1 (pre-survey), 152/485 (31%) at T2, 133/424 (31%) at T3, 99/409 (24%) at T4, 98/395 (25%) at T5, 91/372 (24%) at T6, and 66/350 (19%) at T7. Table 2 provides additional demographic data for participants who consented to having their comments shared for each of the seven time points.

**Table 2:** Demographic profile.

Demographic	T1 (n = 283)	T2 (n = 152)	T3 (n = 133)	T4 (n = 99)	T5 (n = 98)	T6 (n = 91)	T7 (n = 66)
*Occupation*							
Nursing	77	41	41	31	31	29	16
Physician/Resident	29	15	10	*	11	8	10
Laboratory or Medical Technician/Technologist	26	12	7	8	6	6	*
Administrative Personnel	26	12	7	7	6	7	*
Allied Health (Dietician, Social Worker, Speech Language Pathologist)	23	21	11	12	10	*	5
Scientist/Research Personnel	22	13	15	8	7	9	9
Physio/Occupational Therapist	22	8	8	12	8	*	*
Manager (Clinical, Non-clinical, Senior)	15	8	15	*	7	11	7
Patient-facing Admin or Assistants	15	6	5	*	*	*	*
Other Healthcare Personnel	28	16	14	21	12	10	5
*Gender*							
Female	230	128	108	83	81	75	52
Male	50	22	23	13	15	13	11
Other/Prefer not to say	3	2	2	3	2	3	3
*Age*							
Mean (SD)	40 (11.6)	42 (12.0)	41 (10.9)	40 (11.4)	41 (11.3)	42 (11.1)	43 (11.8)
Range	19–75	19–75	23–75	19–75	23–75	19–75	19–75
*Education*							
High school	5	1	0	2	1	2	1
College diploma	51	16	16	7	12	12	7
Undergraduate degree	84	51	41	37	37	27	20
Professional/Graduate degree	143	84	76	53	48	50	38
*Ethnic Group*							
African/Black	12	8	7	3	2	4	2
Asian	76	38	30	30	26	17	14
East Indian	19	8	12	7	6	6	2
European/White	154	90	75	50	59	59	46
Other/Mixed/Multiple	22	8	9	9	5	5	2
*Marital Status*							
Single	91	47	36	35	28	21	17
Married/Common-law	180	97	91	61	67	66	45
Divorced/Separated/Widowed	12	8	6	3	3	4	4
*Vaccination Status*							
Number vaccinated (%)	NA	98 (64.5)	128 (96.2)	95 (96.0)	96 (98.0)	91 (100)	65 (98.5)

cells with values <5 have been marked with *

### Themes

The inductive analysis revealed three themes that contributed to moral tensions and injury: 1) experiencing distress with staffing shortages, increased patient care needs, and visitor restrictions; 2) feeling devalued and invisible due to lack of support and inequities; and 3) polarizing anti- and pro-public health measures and incivility. S1 File provides the coding schema with more narrative examples.

#### Experiencing distress with staffing shortages, increased patient care needs, and visitor restrictions

Over the course of the pandemic, in the hospital, there were fluctuations in staffing with strain due to shortages as well as increased workload and increased volumes of acutely ill (COVID-19 and non-COVID-19) patients. As one nurse shared *“the staffing has gotten a lot worse*, *and it’s become overwhelming* (T5)*”*, while another described their *“workload has increased to a ridiculous demand [with] heavier patients*, *more discharges and admissions every day*, *and short staffed more frequently* (T4)*”*. As a medical imaging technologist (T4) noted *“work is getting more hectic right now as volumes increase and its lack of staffing and the hectic work environment that stresses me”*. The increased workload as a result of staffing shortages and increase in patient volumes and complexity was further exacerbated, particularly for nurses, by having to provide education to redeployed or new staff. Shortages of staff occurred as a result of sick calls, recruitment challenges, and turnover with some participants sharing that nurses were leaving the organization for less stressful environments and better compensation. The following excerpts provide examples of this theme.

*“Working in the ICU has put additional strains with the patient population, limited resources and increased teaching to unqualified staff. There is no financial compensation for nurses. It makes me feel ICU nurses are expendable and worthless. Short staffed or staffed to quota but with unqualified staff puts tremendous strain on the ICU nurse.”* (T3 Nurse)

*“The lack of staff has been incredibly difficult*. *So many sick calls*, *so many short staffed shifts*. *No one is applying for job postings*. *No one wanting overtime because everyone is so burnt out*. *We have yet a chance to breathe despite the numbers*.*”* (T4 Occupational Therapist)

*“Nurses aren’t growing as professionals*. *People have left the unit due to lack of growth*, *difficult assignments*, *toxic social unit environment and other personal reasons*.*”* (T5 Nurse)

*“It’s crystallized my retirement plans and likely will be earlier than pre-pandemic*.*”* (T6 Physician)

Grappling with ongoing staffing shortages and heavy workloads evoked feeling stressed, burnt out, and upset. Some respondents also expressed feeling resentment and betrayal towards other staff that were taking sick time off or were no longer redeployed, with hospital management for not supporting staff more throughout the pandemic. As one nurse (T2) noted “*I frequently feel upset and betrayed by them calling in sick because it makes us short-staffed for the shift*, *which in turn creates more stress on the rest of us who continue to come in to work regularly during this pandemic*.*”* Others expressed feeling morally distressed and guilty about coming to work and not being able to provide quality care or when they themselves had to take a day off work due to being sick. For some respondents, their levels of exhaustion and burnout and managing within a crisis situation were impacting their ability to provide quality care. As a speech language pathologist noted *“we were in a crisis situation*, *making quick decisions that have never before had to be made and those may not have always led the best outcomes but they always came from good intentions from all involved parties* (T3)*”*. This theme is further illustrated in the following quotes.

*“In the second wave patients have been coming in more sick than I can remember. The amount of time spent providing bedside nursing care for just 1 patient is increasing. I feel it is unsafe, for my patients, for myself. I feel taken advantage of, working in increasingly difficult circumstances with added pressure and urgency.”* (T2 Nurse)

*“With staff shortages and ongoing high caseload demands*, *I feel guilt at taking more than 1 day (a SICK day*!*) off work*. *Taking any planned time off is more stressful than being at work*.*”* (T7 Nurse)

*“Due to staffing demands and workload challenges*, *my role has shifted in responsibilities to work that I don’t feel passionate about*, *which created inner conflict for me that is challenging*.*”* (T7 Social Worker)

More severe moral tensions emerged with some participants sharing that they felt they were abandoning their patients when they were redeployed, while others described having to compromise the care they were capable of providing. The toll of visitor restrictions also was observed to impact the care of both the patients and their care partners, as one participant described as *"keeps them up at night”*. This theme is further illustrated in the following quotes.

*“The most difficult part of redeploying to another area of the hospital, was the sense that I was abandoning my own patients.”* (T4 Social Worker)

*“Very difficult to feel you are doing a good job when you are not providing the same amount to therapy that you were able to provide prior to the pandemic*.*”* (T6 Occupational Therapist)

*“What keeps me up at night is the cumulative effect of seeing the isolation and confusion of our patients*, *and their families throughout this pandemic*, *and the struggles they have with understanding what is going on*.*”* (T3 Healthcare Personnel)

*“I find the rules about patient visitors and care partners to be unfair*, *discriminatory*, *and inconsistently applied*. *This is very distressing in the clinical environment*.*”* (T3 Physician)

#### Feeling devalued, unappreciated, and invisible due to lack of support and inequities

Closely aligned with the first theme, feeling devalued and invisible due to lack of support and inequities emerged as further examples of tension and moral distress. This theme captures the feelings evoked from the lack of acknowledgement and response from both the organization’s leadership and government. This is illustrated as one nurse described that *“support for staff working during the pandemic from senior hospital management [and] the government continues to be sporadic and patronizing* (T3)*”*. They further added *“being told verbally by everyone that they so thankful for your service and hard work*, *but then doing nothing concrete (increased pay*, *more resources) is disingenuous and makes me more depressed than if they had said nothing at all”*. Others described feeling unsupported (e.g. having to take vacation days when being off due to a COVID-19 high risk exposure), unappreciated with having to do more with less, and emotional as noted by one nurse “*it has been tiring*, *emotionally draining and feelings of being unsupported by some colleagues and management* (T3)*”*. Further narrative excerpts exemplify this theme.

*“Ongoing staff shortages has caused ongoing stress and burnout. The lack of notice by management or repercussions sets the tone that this is acceptable going forward and they don’t care about us.”* (T4 Healthcare Personnel)

*“There is no flexibility and nurses are not seen as humans*, *just pawns that are replaceable*. *They are tired of being treated like they are unimportant and are leaving in droves*.*”* (T6 Nurse)

Over time, feelings of sadness, disillusionment. irritability, resentment, and apathy were experienced. For example, one healthcare personnel shared that COVID-19 created apathy in their department that resulting in them feeling *“being bad at your job*, *unsatisfied* (T4)*”*. Other narrative examples include:

*“The expectations are high and it feels like management doesn’t really care that we are languishing on the frontlines. I don’t feel the government care about the toll this has taken on us. I feel wrung dry.”* (T4 Occupational Therapist)

*“I feel sad and disillusioned than ever before*. *I feel my manager and hospital just don’t care about protecting staff*.*”* (T7 Nurse)

*“I don’t feel valued by the organization*. *My ability to find my resiliency against the day to day stressors is now lacking and it comes out in my irritability at home too*.*”* (T7 Nurse)

Fewer participants shared a counterview to not feeling supported as they shared their appreciation for what was described as *“small things”*, *“little things”*, and *“small tokens”* that the hospital leadership provided. This included providing free coffee and tea, wellness kits, t-shirts, and acknowledgement of people who exemplified the hospital’s values during open forums and emails as noted in the following quotes:

*“The small things like the wellness initiative, tea robot makes a world of difference.”* (T4 Resident)

*“I appreciate the organizations commitment to supporting and recognizing staff-small tokens of thanks-sleep kits*, *t shirts*, *treats*, *values shout outs*, *town halls*, *daily communication*.*”* (T5 Senior Manager)

Several inequities emerged over time that contributed to feelings of resentment, anger and hurt between various groups: those providing direct clinical service vs. those who did not (e.g. administrative staff, researchers); those seeing patients within the hospital vs. their counterparts in the community; or those receiving extra pandemic pay compared to those who did not. Allocation of personal protective equipment (PPE) and access to COVID-19 vaccines varied between patient facing physicians and staff in the hospital and those that were not patient facing.

For those not who perceived not having the same access to resources or deemed *“essential”* the perception of a *“two-tiered system”* emerged and generated feelings of being left out, unprotected, anxious, invisible, and overlooked. Participants also shared feeling burnt out as a result of not being replaced themselves, while other clinicians (e.g. nursing) were replaced. This theme is further elucidated in the following narrative.

*“I understand that frontline workers are the focus, but demanding auxiliary staff like admins be on site, while not providing PPE or salary supports, makes people feel expendable and unappreciated.* (T3 Administrative Assistant). *The two-tiered system for clinical vs non-clinical staff contributed to my anxiety about getting sick and my sense that the organization did not value me or my work.”* (T4 Administrative Assistant)

*“When surge beds were introduced on the 3rd floor*, *nursing staff was augmented to accommodate the increased work load but not Allied Health staff–*[there was] *mixed messaging from upper management regarding treating staff fairly during these stressful circumstances*.*”* (T4 Physiotherapist)

*“Allied were omitted from pandemic pay even though we had just as much risk*. *We do not feel appreciated or valued*. *Instead of spending money on little gifts we would all prefer to be paid more or a bonus*.*”* (T5 Dietician)

*“Those of us in non-clinical positions lack a lot of emotional support*. *We have to navigate the resources available to us alone*.*”* (T5 Research Personnel)

#### Polarizing anti- and pro-public health measures and incivility

A constellation of emotions also emerged associated with the societal response to COVID-19. Respondents shared it is *“frustrating”*, *“irritating”*, *“demoralizing”*, *“very stressing”*, *“overwhelming”* and *“disheartening”* that there are those in society who are vocal about not believing the seriousness and impact of COVID-19. Particularly, respondents identified those who have protested against public health measures (e.g. wearing masks or face shields, social and physical distancing) or who have travelled during the second wave. For some respondents, this added to their level of fatigue and was *“wearisome”* as they continued their efforts in keeping themselves safe, the patients in the hospital safe, and their family safe. As one respondent noted that it was an *“insult to many of our sacrifices at work and at home”*. Collectively, this has resulted in what is referred to as *“COVID fatigue and burnout and feelings of despair”* and for some *“feeling resentful”*. This theme is further elucidated in the following narrative examples:

*“It remains wearisome—the constant specter of dealing with the unknown and having to adapt to the next challenge, the sense of helplessness and the demoralizing feeling of doing the right thing at personal cost when colleagues and the community are not. I cannot but help feel resentful.”* (T2 Resident)

*“We sacrificed for 1 year and have achieved nothing*. *People are not abiding by the social measure put in place*. *I as a healthcare worker feel insulted by this*.*”* (T3 Healthcare Personnel)

*“It is hard to suppress judgement about people choosing not getting vaccinated*, *or not following public health directives*. *My well of compassion is empty at times*. *Then I feel a sense of failure at myself for feeling that way*.*”* (T6 Nurse)

*“The "freedom convoy protests" have significantly increased my stress level knowing that a portion of the population has no confidence in public health and science*.*”* (T6 Physiotherapist)

*“I find that I’ve been losing confidence in the public’s will to do what is right and to sacrifice a little more for the common good*.*”* (T7 Occupational Therapist)

Over time, some respondents shared increasing difficulty being empathetic and compassionate for those who were not vaccinated and how this was impacting them and their ability to provide optimal care. Further, increasing examples of patient incivility resulting in emotional burnout were provided as one healthcare personnel shared *“patients are back to pre-covid levels of frustration that they take out on staff and I am finding myself more emotionally burnt out as every day that we continue to live through this pandemic feels increasingly Sisyphean”* (T5). Additional quotes illustrate this theme:

*“It’s even more challenging to provide equal care to patients who aren’t cooperative and willing to accept your care. This is the most draining part of my job; working with patients and families who don’t appreciate your care.”* (T4 Occupational Therapist)

*“It’s increasingly difficult for me to have empathy for people who choose their physical comfort at the risk of endangering others*.*”* (T5 Healthcare Personnel)

*“We’re dealing with patients with COVID who are anti-vaxx*. *How do you find compassion for them when you’re so exhausted*?*”* (T5 Nurse)

*“Really nasty families/patients has really made me dislike work and society*.*”* (T6 Physiotherapist)

*“We have many more patient outbursts and episodes of violence than before the pandemic*. *I find myself becoming more weary as time goes on*.*”* (T6 Physician)

*“One of the most stressful parts of dealing with the pandemic has been dealing with visitors who are not compliant with PPE*, *not wearing masks*, *and who are angry with the visitor policies*. *Some interactions have left me shaken and upset*.*”* (T7 Nurse)

Incivility was also experienced on a colleague-to-colleague level as noted by one nurse (T7) who shared *“the main stress that comes from my workplace is not the patient population but from some of my colleagues who are either abusive*, *intrusive*, *and malicious”*.

## Discussion

Our study is one of the first to highlight the impact of COVID-19 on non-clinical staff (e.g. researchers, administrative staff, clinical and non-clinical leaders and directors). In addition to the existence of moral tensions and injury, our study findings elucidate and further unpack how HCWs and non-clinical staff are experiencing moral tensions, distress, and injury associated with the COVID-19 pandemic. For example, the growing staffing shortages coupled with increased workload situated those HCWs and non-clinical staff who remained working in a cycle of stress and burnout that resulted in feeling resentful and being upset culminating in moral distress. This is similar to what has recently been reported around how increased working hours, exhaustion and limited logistical support has eroded HCWs’ well-being [2, 15, 35]. This cycle and feelings experienced were further exacerbated with the moral tension entrenched in one’s duty to care and not being able to provide the care to patients they felt they should receive. Further, our study elucidates HCWs’ duty to care is a deeply held moral value that when challenged has both acute and chronic psychological impact (e.g. the social worker who felt they were abandoning their patients when they were redeployed), which overtime evolved to moral injury. Moral injury also was experienced with clinical and non-clinical staff who bore witness to the devastating impact of visitor restrictions on patients and families.

Similar to other studies, [1, 4, 13–15, 17–20, 24–27, 36, 37] the spectrum of psychological impact of COVID-19 on HCWs that emerged in our study is substantial, including experiencing moral tensions and injury associated with working conditions and less than optimal care juxtaposed with one’s duty to care. For example, one study shared that 41% (134/328) of nurses reported moral injury [2] while another reported that although moral distress had declined, moral injury remained stable over time in the first year of the pandemic [14]. A recent systematic review also revealed that HCWs reported the prevalence of post-traumatic stress symptoms (PTSS) to occur between 2.1 and 73.4% [24]. This review also posited ongoing impact on HCWs who likely will continue to experience acute and chronic unpredictable, occupational stress leading to PTSS [24]. A related measure of secondary traumatic stress (STS), defined as an acute stress reaction derived from caring for others who are suffering or have been traumatized, was reported to occur with 41.3% of HCWs in a recent study [3]. This study also reported the prevalence of STS symptoms was 47.5% with those HCWs working with COVID-19 patients [3]. Further, ongoing exposure to moral distress can also result in the ‘crescendo effect’ where HCWs react more strongly to similar moral distressing stimulation/situations the next time they occur [27, 38].

Our findings add to the growing evidence base around the devastating impact of the pandemic restrictions for HCWs experiencing moral injury when their duty to care is challenged and from observing care that is less than optimal for patients and their family members [11, 13, 14, 25, 27, 28]. Duty to care is not a new concept, however it has gained more attention amid the COVID-19 pandemic [11, 12, 14]. Similar to what was reported in our study, it is well known that HCWs often will work longer hours and more shifts and sacrifice personal desires (e.g. time away from loved ones) for the benefit of patients [12, 14]. Our study also included examples of duty to care whereby people came to work despite knowing the clinical area would be short staffed to support their colleagues, often at the expense of their well-being. The tension around not being able to provide optimal care or bearing witness to sub-standard care is similar to what other studies found [11, 13, 14, 27, 28, 39]. One study reported that nurses were overwhelmed with sadness being exposed to unimaginable pain and suffering and bearing witness to the realities of COVID-19, including death and feeling ineffective [11]. For some nurses, this evoked a stronger duty to care, while for others they decided to leave the organization and the profession [11]. As in our study, feeling that ineffectual care and the inability to enable therapeutic family presence due to visitor restrictions was shared as sources of stress amongst HCWs in other studies [13, 14, 39]. Our study finding is also echoed in literature around STS arising when rescue care-taking efforts are not successful and HCWs observe patients’ physical pain, psychological suffering, and death [3, 40].

This is one of the first studies that elucidates the perceived unequal access to resources and support between clinical and non-clinical staff, within different clinician disciplines (e.g. pandemic pay); staff working in clinical areas providing care to COVID-19 patients and staff that are not. The inequitable access to resources in our study likely impacted HCWs’ and non-clinical staff’s level of stress as previous research found PTSS was more likely to be experienced when there is a lack of social support [14].

The feelings of betrayal and resentment towards the organization’s leadership and government that emerged in our study is consistent with recent studies [2, 26–28]. One study found during a time where support from leadership was most needed the exact opposite occurred, perpetuating feelings of betrayal, pain, frustration and anger [26]. In our study, feelings of apathy due to lack of perceived support emerged and is echoed in another study that reported resident physicians had developed a more detached attitude towards their work [15]. The constellation of emotions emerging from societal response adds further insights into the moral tensions and injury HCWs and non-clinical staff are experiencing as a result of the pandemic.

Our study elucidates the tensions between enacting (or not enacting) public health measures and the devastating impact on HCWs’ and non-clinical staff’s psychological well-being. Moral injury often occurred when care providers experienced conflict in providing optimal, empathetic and compassionate care to patients who were unvaccinated or uncivil. Other research has reported a significant moral burden for HCWs caused by feeling betrayed by leaders within their organization [2].

Urgent efforts are required to prevent and mitigate the detrimental impact of moral tensions and injury amid our healthcare workforce [14, 24, 25]. These efforts need to span the spectrum of moral distress, tensions, and injury that balances duty to care as well as worker well-being [12], likely requiring different strategies for related psychic sequelae [14, 24]. Underpinning these efforts requires a focus on enhancing resilience at both an individual level [14] and collective level [41] that ensures a focus beyond individual accountability for managing stress and resilience. In terms of enhancing individual resilience, a recent Cochrane review delineated the following resilience factors: having a strong sense of purpose, ability to adapt and cope, positive mental state, confidence, optimism and perceiving strong social support [42]. This review also reported that resilience training improved measures of resilience and acutely reduced symptoms of depression and stress [42].

In addition to resilience, training and education needs to include how to anticipate and manage the effects of traumatic exposure and how to support each other during times of moral injury [24]. Organizations can further bolster resilience and alleviate the long-term consequences of COVID-19-related stress on the healthcare workforce’s mental health [14] by offering services and programs that address work–life balance, as well as promote connectedness and mutual support [15, 43]. A recent scoping review recommended protective factors at the individual level (a belief in a just world and self-worth) and at the social level (forgiving environment) [27]. In one study, a supportive work environment was found to be a protective factor against negative psychological health and PTSS among HCWs during the COVID-19 pandemic [44]. In another study, nurses explicitly outlined the following resources and other support that would have been helpful during the COVID-19 pandemic: 1) counseling or other emotional support; 2) peer support (formal or informal); 3) education and ethics support; 4) wellness offerings; and 5) spiritual or faith support [2]. This study also identified the need for a stronger organizational infrastructure (availability of resources; clear communication; offering hazard pay and other incentives; and consistent enforcement of policies, practices, and rules) to support HCWs during COVID-19 [2].

Finally, efforts also are needed to enhance collective moral resilience, defined as a shared capacity arising within a group with mutual trust and connectedness, through the process of sharing ethically challenging situations, thinking together about the challenges, and dialogue to sustain or restore moral integrity in response to moral suffering [41]. Hosting communities of practice, whereby collective moral resilience and ethical practice environments are created by focusing on mitigating solutions to system and culture issues that are contributing to moral distress and occupational burnout, is a strategy for organizations to consider [41].

Further, providing programs and resources to bolster mental health resilience in our healthcare workforce is paramount [4, 42, 45]. For example, a multi-pronged approach involving a range/variety of strategies to build and maintain resilience emerged from a recent scoping review, and included providing information, psychosocial support and treatment; monitoring the health status of healthcare workers; and using various forms and content of psychosocial support (e.g. encouraging peer support, sharing and celebrating successes) [5, 7].

### Limitations

The following limitations need to be taken into consideration. Although the method of recruitment initially went out to all hospital employees, physicians, learners, retail employees, and contractors, there was not a sampling strategy. Further, the decreased sample over time and those who did not participant initially or chose to stop participating may not be a representative sample. Self-selection and social desirability biases may also have been present.

## Conclusion

The spectrum and severity of the emotional, psychological, and physical stress leading to moral injury experienced by the healthcare workforce that emerged in this study is concerning. Moral injury was experienced by HCWs and non-clinical staff as they grappled with the tensions between less than ideal working conditions, juxtaposed with their duty to care, and feeling betrayed by their leaders and society, while losing compassion and empathy for those who chose not to enact public health measures. Our findings point to continued, renewed, and new efforts in enhancing both individual and collective moral resilience to mitigate current and prevent future moral tensions and injury. There is no one size fits all strategy, rather different strategies are required to reconcile the current tensions between duty to care and worker well-being across the spectrum of moral distress and injury.

S1 File provides the coding schema with more narrative examples.

## Supporting information

S1 File (DOCX)
