# Supplementary material for: Long-term impact of COVID-19 pandemic: Moral tensions, distress, and injuries of healthcare workers
Source: PLoS One. 2024 Sep 27;19(9):e0298615. doi: 10.1371/journal.pone.0298615 (PMC11432829; doi:10.1371/journal.pone.0298615)
Supplement: S1 File — (DOCX) [file pone.0298615.s001.docx]

Supplementary File 1

Coding Schema

| Themes | Narrative Examples |
| --- | --- |
| Experiencing distress with staffing shortages, increased patient care needs, and visitor restrictions | *“In the second wave specifically, patients have been coming in more sick than I can remember in the past year. The amount of time spent providing bedside nursing care for just 1 patient is increasing. I feel it is unsafe, obviously for my patients, but also for myself. I'm taking shorter breaks, and taking breaks later in the day. Eating and drinking less throughout my shift. And given recent changes on unit x, it is only going to get worse. I feel taken advantage of, working in increasingly difficult circumstances with added pressure and urgency. At the end of the day, what can nursing staff do? Abandon our patients and colleagues?”* (T2 Nurse)  *“What keeps me up at night is not a single episode or set of events at the hospital, but the cumulative effect of seeing the isolation and confusion of our patients, and their families throughout this pandemic, and the struggles they have with understanding what is going on, and being certain that they are able to communicate clearly.”* (T3 Healthcare Personnel)  *“I find the rules about patient visitors and care partners to be unfair, discriminatory, and inconsistently applied. This is very distressing in the clinical environment.”* (T3 Physician)  *“I feel working in the ICU has put additional strains with the patient population, limited resources and increased teaching to unqualified staff. There is no financial compensation for nurses and yet doctors are reaping financial rewards that are extreme. It makes me feel ICU nurses are expendable and worthless. Short staffed or staffed to quota but with unqualified staff and somehow this is acceptable. Puts tremendous strain on the ICU nurse.”* (T3 Nurse)  *“The lack of staff has been incredibly difficult. So many sick calls, so many short staffed shifts. No one is applying for job postings. No one can be hired. No one even wanting overtime because everyone is so burnt out. The effects of the pandemic are far from over in hospital. Our extra beds are still full. We have yet a chance to breathe despite the numbers. We are now playing catch up.”* (T4 Occupational Therapist)  *“The most difficult part of redeploying to another area of the hospital, was the sense that I was abandoning my own patients.”* (T4 Social Worker)  *“The biggest challenge I'm finding currently is that I face moral dilemmas of wanting to provide great care for my patients however the workload demands make that challenging therefore I'm having to compromise the type of care that I know I'm capable of providing.”* (T4 Occupational Therapist)  *“Will always attempt to push through for the benefit of a patient at a detriment to self-so when the organization asks more of you, even when you know you can't, you do it because you don't want to let the patients down-not their fault the system stinks! I have guilt if I don't give each patient "the best care" guilt and high standards drives my work at this point, rather than a true compassion like pre-pandemic. I can't leave work knowing I didn't give it my best go, given our work impacts lives.”* (T4 Occupational Therapist)  *“The nursing shortage is having an extreme effect on the quality of work I am able to do. I worry about coming into work and being short staff. Leadership says nothing to us about being short staff. Doctors make snide remarks about how short we are and it is upsetting because some of us come to work despite feeling at our ropes end. This is the first time I've called in sick in over a year because I am just so tired of it all.”* (T4 Nurse)  *“I still feel betrayed by the hospital and had contemplated quitting many times despite "being short of RN's". However, it is with my clients and the meaningful work we do that convinced me to stay.”* (T5 Healthcare Personnel)  *“The staffing has become more and more short on the unit and it's been hard to pick up extra assignments due to lack of nurses. Patients are getting sicker and need more complex care.”* (T5 Nurse)  *“Nurses aren't growing as professionals. People wants to leave and are seeking other jobs elsewhere. People have left the unit due to lack of growth, difficult assignments, toxic social unit environment and other personal reasons.”* (T5 Nurse)  *“It's crystallized my retirement plans & likely will be earlier than pre-pandemic.”* (T6 Physician)  *“We are continually given more work to do, with no extra staff. Not able to take time off in case of staff shortages. My mental health has never been so poor. When I am off for the weekend, I don't want to do anything (even if we could). Feeling very used and abused during this pandemic.”* (T6 Pharmacy Technician)  *“Very difficult to feel you are doing a good job when you are not providing the same amount to therapy that you were able to provide prior to the pandemic.”* (T6 Occupational Therapist)  *“With staff shortages and ongoing high caseload demands, I feel guilt at taking more than 1 day (a SICK day!) off work. Taking any planned time off is more stressful than being at work, since we have to prepare for/make up for the work we missed.”* (T7 Nurse)  *“Due to staffing demands and workload challenges, my role has shifted in responsibilities to work that I don't feel passionate about, which created inner conflict for me that is challenging.”* (T7 Social Worker) |
| Feeling devalued, unappreciated, and invisible due to lack of support and inequities | *“It feels like nurses are less appreciated now than at the beginning of the pandemic. The staffing is worse and we are expected to do more with less.”* (T3 Nurse)  *“I understand that frontline workers are the focus, but demanding auxiliary staff like admins be on site, while not providing PPE or salary supports, makes people feel expendable and unappreciated. What happened in practice was a two-tiered system that left many "essential" workers feeling left out and unprotected.”* (T3 Administrative Assistant)  *“I feel as though x does not invest in it's own staff aside from clinicians. This includes paying staff a fair wage that is aligned with costs of living and inflation, as well as providing professional development. A significant portion of x staff are not clinicians, but are overlooked.”* (T3 Researcher/Scientist)  *“The expectations are high and it feels like management doesn't really care that we are languishing on the frontlines. I don't feel like management or even the government care about the toll this has taken on us. They want blood from a stone, I feel wrung dry. I have never in my life played the lottery so much than I have throughout the pandemic... just the dream of getting away from it all, having no financial worries so I wouldn't have to keep dealing with the stress of work.”* (T4 Occupational Therapist)  *“Feel as though management has not supported staff. There are ongoing staff shortages (despite the COVID-19 crisis being managed) has caused ongoing stress and burnout. The lack of notice by management or repercussions in this behaviour/performance sets the tone that this is acceptable going forward and they don't care about us.”* (T4 Healthcare Personnel)  *“Management has done next to nothing to address it. It is demoralizing. We are being told time and time again to prove how hard we are working rather than anyone actually believing us, as we limp through the day and sick calls are at an all-time high. Statistics being collected count numbers only, and don't account for acuity.”* (T4 Nurse)  *“I was being told I was essential enough to have to be in the office everyday, but not essential enough to be given adequate PPE or pandemic pay. The two-tiered system for clinical vs non-clinical staff really contributed to my anxiety about getting sick and my sense that the organization did not value me or my work enough to fight for the benefits and protections clinical staff were given. If staff are deemed "essential" they should all receive the same protection and benefits. Outbreaks in hospital settings are common and claiming only clinical staff were at significant risk was neither accurate nor fair.”* (T4 Administrative Assistant)  *“When surge beds were introduced on the 3rd floor, nursing staff was augmented to accommodate the increased work load but not Allied Health staff - mixed messaging from upper management regarding treating staff fairly during these stressful circumstances (and hence, contributing to staff burnout for those not supported).”* (T4 Physiotherapist)  *“The wage freeze from the Ford government reinforced my feelings of being unappreciated for my work.”* (T5 Nurse)  *“We have been experiencing a nursing shortage which has added another layer to what we are dealing with from COVID. The hospital doesn't seem to be doing enough to support us. Senior nurses are leaving in large numbers leaving the units with novice nurses who require lots of training and support. I'm currently looking for another job because I don't feel valued by x anymore. I've been there 10 years so this is a shame.”* (T5 Nurse)  *“It felt like after the first year of the pandemic, people started to care less about healthcare professionals. We went from feeling very appreciated, to protests outside the hospital and feeling like we don't have support from the government or the people we serve.”* (T5 Nurse)  *“Allied were omitted from pandemic pay even though we had just as much risk. We do not feel appreciated or valued. Instead of spending money on little gifts we would all prefer to be paid more or even a bonus like Providence did where they gave each of their staff 100 dollars.”* (T5 Dietician)  *“Working in a community clinic, I felt we did not have any support from the main hospital. We were casted aside for pandemic pay, to which this day, I still have not received any compensation. We weren't given enough proper PPE.”* (T5 Healthcare Personnel)  *“Those of us in non-clinical positions lack a lot of emotional support. We have to navigate the resources available to us alone.”* (T5 Research Personnel)  *“There is no flexibility and nurses are not seen as humans, just pons that are replaceable. There is not a shortage of nurses, people are coming to terms that they are tired of being treated like they are unimportant and are leaving in droves.”* (T6 Nurse)  *“Too much demand from work but no reward no appreciation no increase in pay scale I feel worthless to be a nurse.”* (T6 Nurse)  *“It's crystallized my retirement plans & likely will be earlier than pre-pandemic.”* (T6 Physician)  *“Lack of support and recognition from management and hospital is very discouraging and disheartening. Violence towards staffs in ED has risen drastically but very little or nothing has been done, no actions or policies have been placed for pts who verbally or physically abused staff.”* (T6 Nurse)  *“I feel more sad and disillusioned than ever before. I feel as though my manager and hospital just don't care about protecting staff.”* (T7 Nurse)  *“I don't feel valued by the organization in ways that I can no longer ignore (pay discrepancies that have been promised to be reviewed for two years, lack of transparency and communication about roles and developing programs). My ability to find my resiliency against the day to day stressors is now obviously lacking and it comes out in my irritability at home too.”* (T7 Nurse) |
| Polarizing anti- and pro-public health measures and incivility | *“It remains wearisome nonetheless - the constant spectre of dealing with the unknown and having to adapt to the next challenge, the sense of helplessness and the demoralizing feeling of doing the right thing all the time at personal cost when colleagues and the community at large are not. Despite all the things I am grateful for and the empathy I try to consider, I cannot but help feeling resentful.”* (T2 Resident)  *“I feel that the second wave was quite a bit harder. Not only at work. I felt that this time there was more anger amongst the public, more disconnected and misguided views/perceptions, and more people who didn't understand that we are constantly trying to find a balance between lowering the rates of infection and the effect of infection control measures.”* (T2 Nurse)  *“The increasingly loud protestations of COVID deniers, anti-maskers, ant-vaxxers, and other prescribers to conspiracy theories has had a negative impact on my perception of society and the public as a whole. I find it very stressing to come across these individuals and have given up trying to educate, inform and converse with them. I believe that they are adding yet another layer of unnecessary stress to our already stressful lives. In addition to these individuals, those that flout the lockdown guidelines, most particularly those in government and other positions of power who travelled, went on vacation, and ignored the lockdown to vacation out of country/county while telling their constituents to stay at home and not see family was incredibly damaging to public perception and has also negatively affected my perception of our society.”* (T2 Nursing Personnel)  *“I am more nervous about how life will be after the pandemic is done as I have been very focused on work and don’t know how I will make time for socializing and other activities that are allowed in normal times.”* (T2 Researcher/Scientist)  *“Everyone involved in healthcare right now feels like they are running on empty and that we are putting out fires that could have been prevented by healthy public policy and our provincial government listening to public health experts. It's upsetting to see the amount of preventable death and loss in the third wave. It is beyond infuriating.”* (T3 Nurse)  *“We sacrificed for 1 year and have achieved nothing. People are not abiding by the social measure put in place. I as a healthcare worker feel insulted by this. I've done nothing for the last 14 months.”* (T3 Healthcare Personnel)  *“I get really frustrated and upset at the anti maskers and people who think it's fake and in turn are making this go on longer. I am scared as I see more people my age come through for my friends and family who aren't yet vaccinated. I have always been positive but there are times that it's hard when you just see so much suffering or fighting on social media etc.”* (T3 Patient-facing Assistant)  *“It's been difficult to adjust to all of the restriction changes outside of work. I feel angry and frustrated that there are so many people who do not care about social distancing and wearing masks indoors.”* (T4 Nurse)  *“It's even more challenging to provide equal care to patients who aren't cooperative and willing to accept your care. I find this is the most draining part of my job; working with Pts and families who don't seem to appreciate your care and efforts.”* (T4 Occupational Therapist)  *“Healthcare has been a thankless job during the pandemic. Families, patients and the hospital itself all expect more from us without increasing staffing or wages. I do not feel fulfilled or valued.”* (T4 Physiotherapist)  *“The issues with the 4th wave in Alberta and Sask. are causing me some emotional distress and irritability, especially now that we are taking patients from Sask, and I am fairly upset that the first one we have received is a rather well known anti vaxxer/COVID conspiracy theorist, and am a bit resentful of this. Also, while I generally do believe in individual choice, I am quite irritable about those fighting against getting the vaccine, especially those in health care, because I do feel as a health care provider, we have an obligation to protect the vulnerable under our care too. Besides, being vaccinated for things such as hepatitis, and the standard vaccinations are an expectation anyways, so why not COVID? How can you be a health care professional and not believe in the science of vaccines and research? Sometimes these feelings of irritation and frustration feel just overwhelming.”* (T5 Nurse)  *“I think the world is more stressed so I worry about the way patients/the public are treating health care workers. People are behaving badly with verbal and sometimes physical abuse against nurses etc. We have been working non-stop for 18 months, gearing up in PPE, risking lives to care for patients and are now being abused by them.”* (T5 Physician)  *“As the general public attempts to return to some form of "normalcy", it's increasingly difficult for me to have empathy for people who choose their physical comfort at the risk of endangering others like myself who are at higher risk.”* (T5 Healthcare Personnel)  *“In recent months, I have found it a bit difficult to work with patients with covid 19 who chose not to get vaccinated and were admitted to our ICU. It is sometimes hard to put aside any feelings you have about this and continue to provide optimal care.”* (T5 Occupational Therapist)  *“At this point, we're dealing with patients with COVID who are anti-vaxx. How do you find compassion for them when you're so exhausted from it all, and have been dealing with COVID since the beginning? Sometimes I think to myself, ‘they did this to themselves’.”* (T5 Nurse)  *“I am exhausted, both mentally and physically. I feel very judgmental toward patients who have chosen to not get their Covid vaccines (because the majority of the pregnant patients who get really sick from Covid are NOT vaccinated).”* (T6 Nurse)  *“It is hard to suppress judgement about people choosing not getting vaccinated, or not following public health directives. I feel like my well of compassion is empty at times. Then I feel a sense of failure at myself for feeling that way.”* (T6 Nurse)  *“I want the pandemic to be over. I wish people would just listen and get vaccinated, instead of making it about their "rights and freedom" or "Them vs. Us". Because we're literally talking about people's lives here. If we can't care about others and only think about ourselves, then we have certainly lost humanity.”* (T6 Nurse)  *“The "freedom convoy protests" have significantly increased my stress level knowing that a portion of the population has no confidence in public health and science.“* (T6 Physiotherapist)  *“I find that I've been losing confidence in the public's will to do what is right and to sacrifice a little more for the common good. Perhaps this is frustration for 2 years of strict restrictions, or envy that other locations have loosened restrictions a long time ago - but with their own accompanying consequences.”* (T6 Physician)  *“There is an immense amount of workplace violence in the ED.”* (T6 Nurse)  *“Really nasty families/patients has really made me dislike work and society.”* (T6 Physiotherapist)  *“I have also found that working in the emergency department, we seem to have many more patient outbursts and episodes of violence than before the pandemic. This rise in violence probably has a few contributing factors, but it is alarming to say the least. I find myself becoming more weary as time goes on.”* (T6 Physician)  *“I feel very torn in the current public health approach to the COVID-19 pandemic. On one hand, a seemingly large majority of people want all restrictions to end and life to return "back to normal."* (T7 Physician)  *“I am losing what little faith I had left in humanity. I don't know how to explain to people that they may be anxious to get back to the way things used to be, but they cannot do that without acknowledging that it affects the millions of us who cannot.”* (T7 other)  *“I find that I've been losing confidence in the public's will to do what is right and to sacrifice a little more for the common good. Perhaps this is frustration for 2 years of strict restrictions, or envy that other locations have loosened restrictions a long time ago - but with their own accompanying consequences.”* (T7 Occupational Therapist)  *“One of the most stressful parts of dealing with the pandemic has been dealing with visitors who are not compliant with PPE, not wearing masks, and who are angry with the visitor policies. I do not like confrontation and the nurses have had to deal with a lot of hostile patients and visitors. Some interactions have left me shaken and upset.”* (T7 Nurse) |
